# Supplementary figures and images for: Behavioral engagement facilitates auditory neuron responses beyond their receptive fields
Source: PLoS Biol. 2026 Mar 13;24(3):e3003707. doi: 10.1371/journal.pbio.3003707 (PMC13012619; doi:10.1371/journal.pbio.3003707)

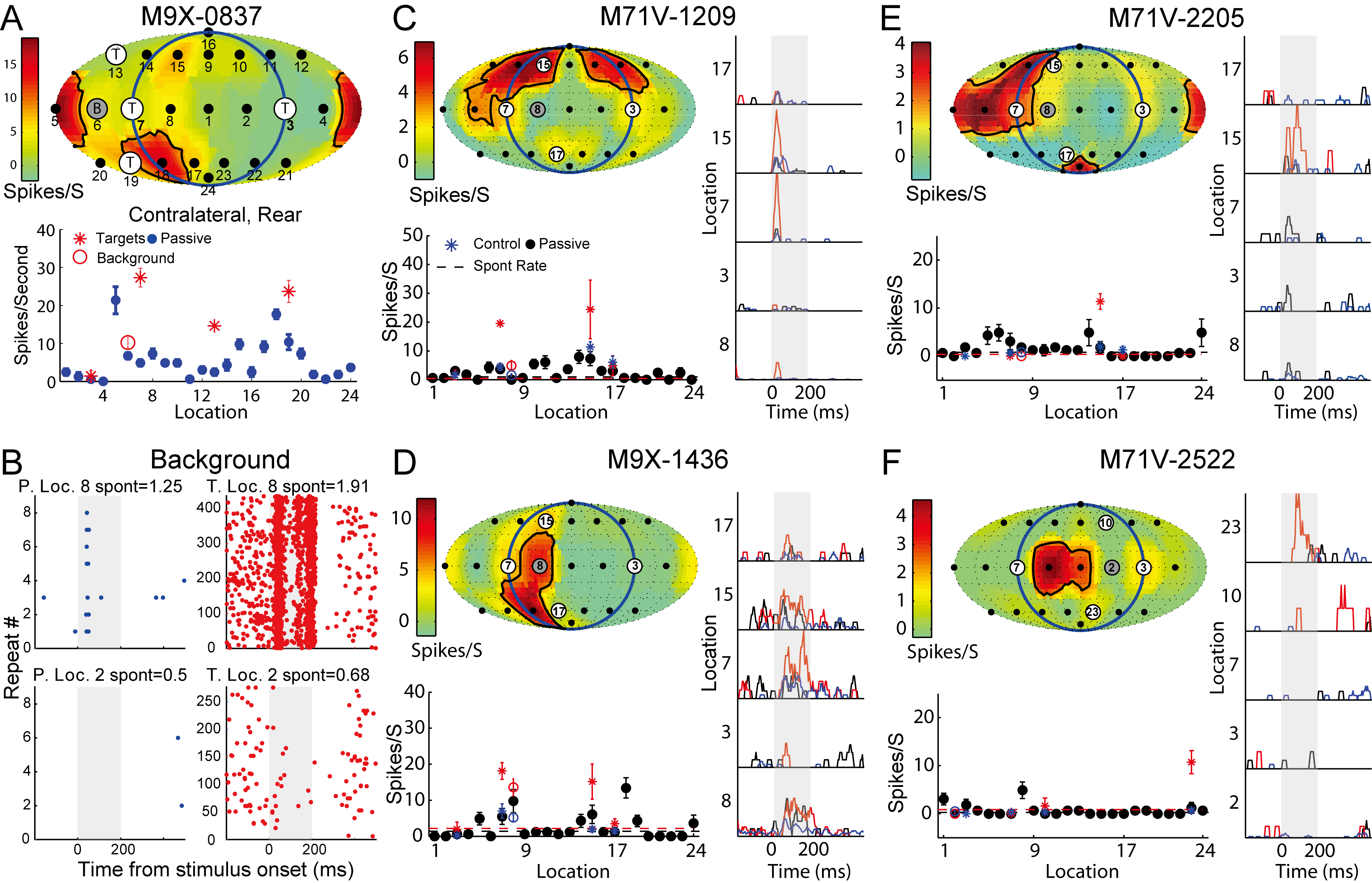

Supplement: S1 Fig — (A) An example session (same unit in Fig 2A) where the background location (#6) and two target locations (#13 and #19) were located at the rear of the animal. (B) Spike raster from two example units (Fig 2A and 2C) at the background locations (#8 and #2) under passive (left) and target (right) conditions. Notice there were many more trials under the target locations. (C–F) Four more example units. Here, we used black, blue, and red colors to represent 24 locations during passive, control, and behaving conditions (five targets/background locations), respectively. Spontaneous firing rates were indicated with colored dashed lines. Data underlying this Figure can be found in S2 Data. (TIF) [file pbio.3003707.s001.tif]

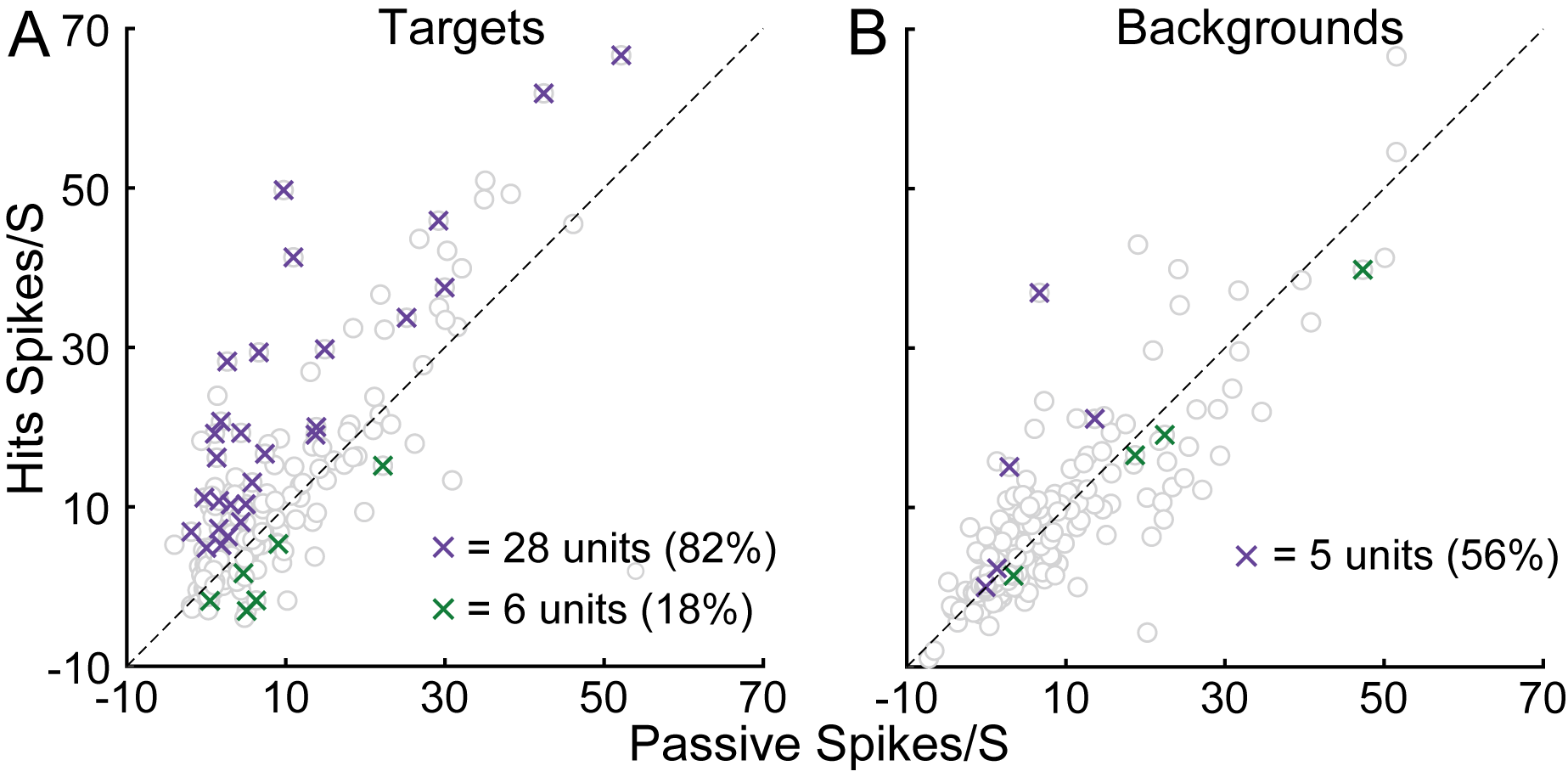

Supplement: S2 Fig — (A) Comparison of firing rates in hit versus passive conditions, averaged across locations and sessions for each unit (n = 208). Gray circles represent nonsignificantly modulated units (99 above and 74 below the diagonal). (B) Same as in A but for background locations. Gray circles indicate nonsignificantly modulated units (97 above and 101 below the diagonal). Data underlying this Figure can be found in S2 Data. (TIF) [file pbio.3003707.s002.tif]

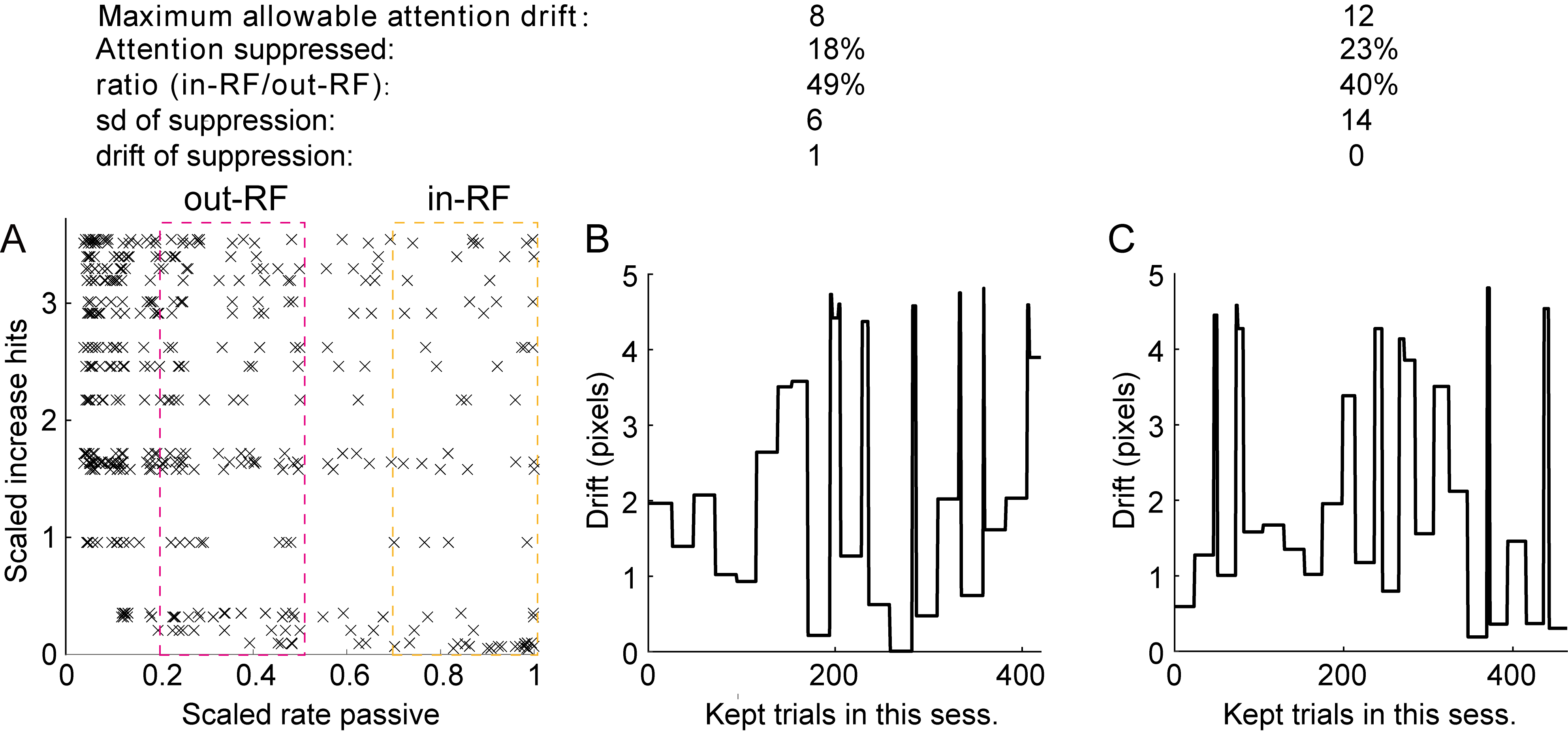

Supplement: S3 Fig — (A) Firing rate during task plotted versus passive firing rate for all data points that were larger than 1, both scaled by the maximum firing rate in the passive condition. The scaled passive rates between 0.2 to 0.5 and 0.7 to 1 were considered as outside and inside of receptive fields, respectively. (B, C) The drift of attention fields in all kept trials from two example sessions. The maximum drifts were only 5 pixels in the kept trials, although the allowable drifts were much larger. Data underlying this Figure can be found in S2 Data. (TIF) [file pbio.3003707.s003.tif]

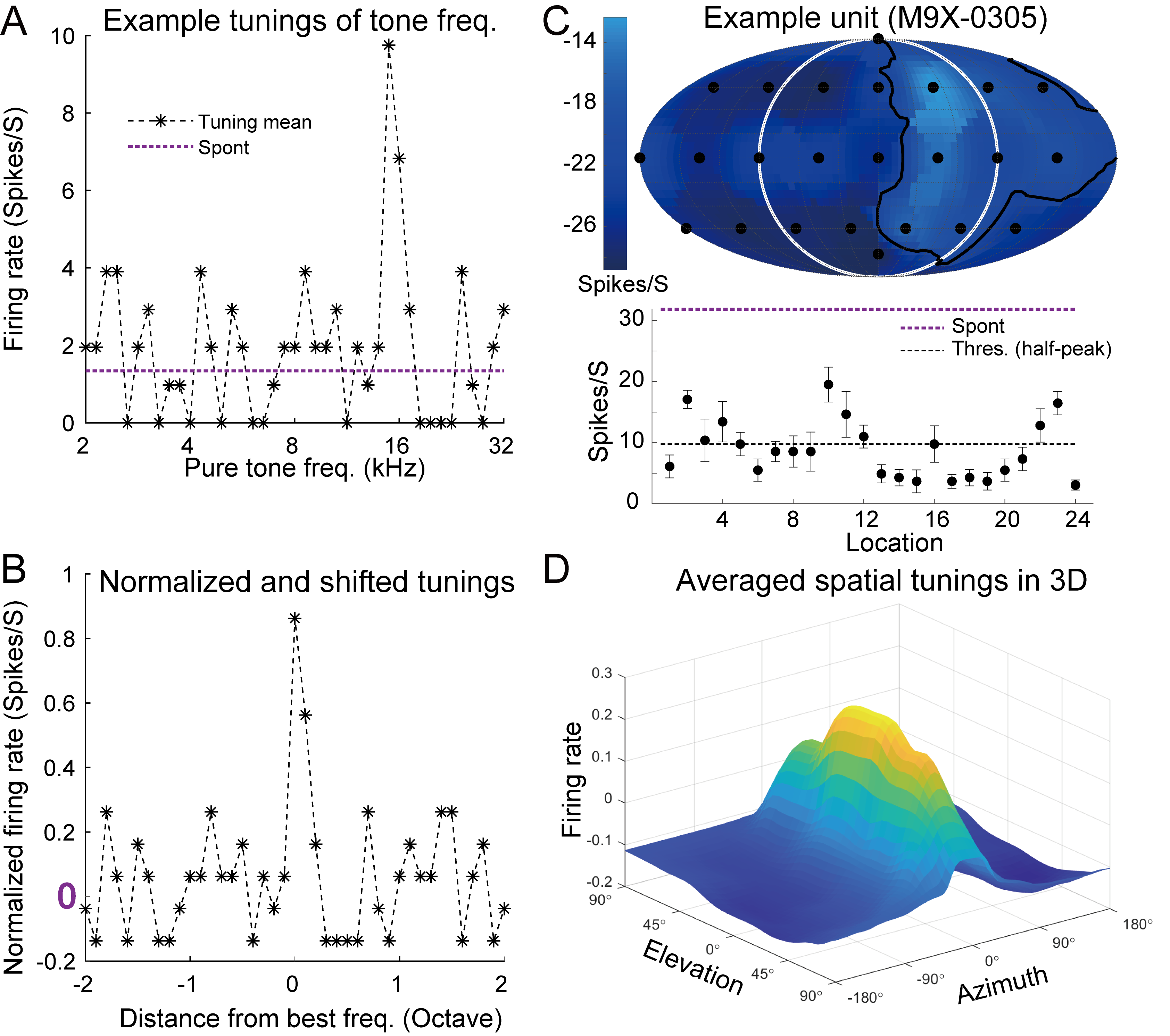

Supplement: S4 Fig — (A) Averaged neural firing rates to 31 sound frequencies (4 octaves, 8 stimuli per octave) of an example unit. (B) The tuning curve was normalized by the maximum firing rate at 16 kHz (the spontaneous firing rate equated to “0”). It was further circularly shifted so that there were 15 frequencies (2 octaves) at both the left and right sides of the peak firing rate. (C) An example unit that was suppressed at all 24 sound locations. Notice that the neuron was still significantly tuned to sound locations (ANOVA, p < 0.01). (D) A 3D view of averaged spatial receptive fields with both color and height at the third axis represented the firing rate. Data underlying this Figure can be found in S2 Data. (TIF) [file pbio.3003707.s004.tif]

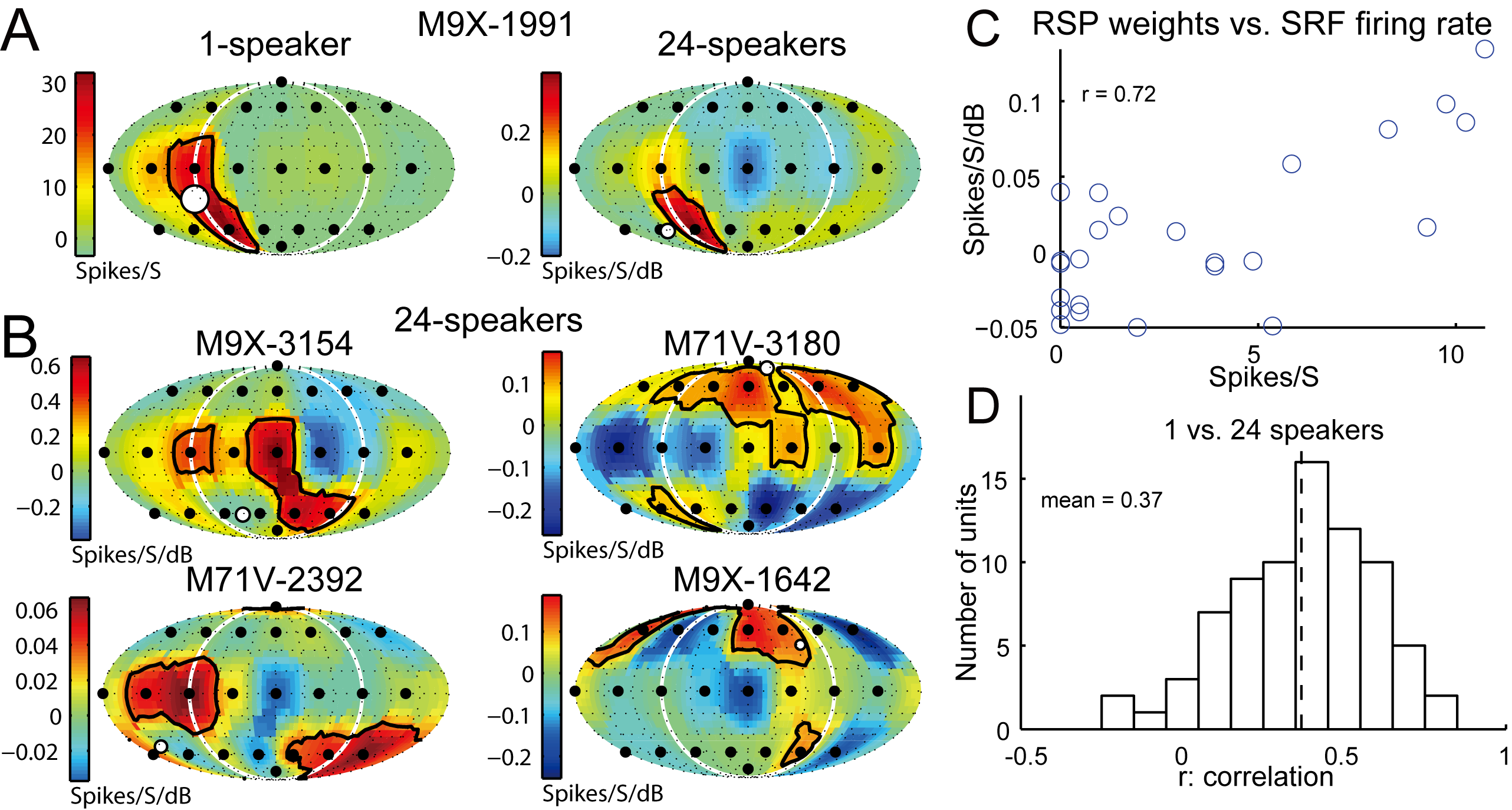

Supplement: S5 Fig — (A) An example unit showed consistent spatial receptive fields under two stimulus paradigms. The position and size of white dots indicate the center of receptive fields and their tuning selectivity, respectively. (B) Four more example units all showed suppressed firing rates at more than one sound location using the RSP stimuli. (C) The scatter plot (X-axis: 1-speaker, Y-axis: 24-speakers) of neural activities at 24 sound locations under two stimulus paradigms. The correlation (r) of neural activities was 0.72. (D) The histogram of correlation for all 77 units. Data underlying this Figure can be found in S2 Data. (TIF) [file pbio.3003707.s005.tif]
