# Supplementary material for: Behavioral engagement facilitates auditory neuron responses beyond their receptive fields
Source: PLoS Biol. 2026 Mar 13;24(3):e3003707. doi: 10.1371/journal.pbio.3003707 (PMC13012619; doi:10.1371/journal.pbio.3003707)
Supplement: Attachment — Submitted filename: Response to Reviewers_v2.docx [file pbio.3003707.s010.docx]

**Responses to Reviewers’ Comments (MS# PBIOLOGY-D-25-03305R1)**

We thank the three reviewers for their constructive and helpful comments. In the revised manuscript (both clean and track-change versions), we have carefully addressed all concerns raised by the reviewers. We hope our responses and revisions have adequately addressed all concerns by the reviewers and would be happy to address any remaining questions. The following is a point-by-point list of all changes made in response to the comments by the three reviewers. The reviewers’ comments are cited (marked in **BLUE** font), followed by our responses. Text copied from the manuscript is highlighted in **RED** font.

**List of new figures:**

**Figure 2A**. Spatial RF, one background location, and four target locations

**Figure 2B** (left). Peri-stimulus time histogram at target location #7

**Figure 4**. Context-independent but task-performance-dependent firing rate modulations

**Supplementary Figure 2.** Attention increases firing rates at the single-unit level

**List of changes on figure numbers:**

The original **Figures 1A-C** have been removed due to copyright concerns

The original **Figures 1D, E,** and **F** have been renamed as **Figures 1C, B,** and **A**

The original **Figures 2A-C** have been renamed as **Figures 1D-F**

The original **Figure 2E** has been renamed as **Figure 2B** (right)

The original **Figures 2F-H** have been renamed as **Figures 2C** and **D** (left and right)

The original **Figure 4** has been renumbered as **Figure 5**

The original **Figure 5** has been separated and renumbered as **Figures 6** and **7**

The original **Supplementary Figure 2** has been moved to **Figure 4A**

Reviewer #1: General Comments:
This is an awkward report of the effects of behavioral task on spatial receptive fields in early auditory cortical areas in the non-human primate, the marmoset.  There are several strengths here, mainly the effects of the behavioral task/attention on these neural responses, and the large spatial region (very close to spherical) tested, so these data should become part of literature as it will be of interest to many.  The primary result is that the behavioral task will influence the response to stimuli in the classically measured receptive field, usually by increases but less commonly by decreases in activity. On the down side, figures 1 and 2 could use some serious work, and while supportive, figures 3 - 5 make only minor contributions to the overall impact of the study, but are still worthwhile in the grand scheme of things.  Fortunately, all these are salvageable but will take some effort on the author's part.  The paper is also fraught with mis-statements, grammatical
errors, etc., of which this reviewer gave up about half way through in noting in 'minor' comments.  A serious re-view and re-editing of the entire manuscript with an eye to the details will make the manuscript much less distracting

We thank the reviewer for recognizing the significance of our work and for your careful reading of our manuscript and constructive comments! We apologize for the errors in the manuscript! In addition to addressing all the concerns listed below, we have thoroughly revised the manuscript, especially in the Results and Discussion sections. We have also carefully checked and corrected any grammatical errors. All the changes can be seen in the ‘track-changes’ version of the manuscript.

Major:
"Dynamic spatial responses, in
146 particular outside RFs, could support functions such as auditory streaming and the cocktail-party
147 effect, and possibly account for the absence of an ordered cortical map of auditory space
148 (Middlebrooks, 2021)."

There is no support for this statement in the results provided and it should be omitted.

We have deleted this sentence from the Introduction.

Figure 1 provides more confusion than clarity and the reference to this figure in the text is inaccurate.

We acknowledge that we referred to the wrong figure panels regarding previous sensory attention tasks. We have removed the original Fig. 1A–C, and the original Fig. 1D, E, and F have been reorganized as Fig. 1C, B, and A in the revised manuscript.

One work around is to show 1D, the behavioral paradigm,

This is now shown in Fig. 1C.

and improve the apparatus figurine to be 3D and more accurately reflect the speaker locations relative the a (stylized) monkey (something similar to the Middlebrooks paper).

This is now shown in Fig. 1B.

This could be placed near the beginning of the results and referred to there while directing the reader to the Methods section for more details.

We have placed Fig. 1 at the beginning of the Results section.

Figure 1. Full-field sound localization task and example neural responses. Speakers were positioned 1 m from the center of the chamber, which had internal dimensions of 2 × 2 × 2 m. The vertical shaded gray bar indicates 200-ms sound stimulus.

Figure 2 is a bit confusing as it is not logically arranged in different panels.

Splitting it into two different figures or re-arranging the panels would probably help.

We have moved the original Fig. 2A–C to the new Fig. 1D–F. We have also rearranged the remaining panels of Fig. 2.

Figure 2. Firing rates increase at locations outside the RFs during spatial discrimination. The modulation index (MI) quantifies the firing rate (FR) difference between the two conditions (target minus passive), scaled by the combined responses (target plus passive).

Figure 2: panels E and H are very compelling. MI for these two cells at these locations should be provided so that the ready gets some indication of what the MI means.

We agree and have added the MI values for these two units to the revised Fig. 2B and D.

We also explained how to calculate the MI in the Fig. 2 legend:

The modulation index (MI) quantifies the firing rate (FR) difference between the two conditions (target minus passive), scaled by the combined responses (target plus passive).

Line 270: should probably read "profiles"

We have revised this section title to read:

Attending to target locations shifts spatial response profiles

Minor:

Line 73 "azimuth and horizontal axes" makes no sense.  Is it supposed to be "azimuth and elevation axes"?

Thank you for pointing this out. We have fixed it as follows:

…, one needs to first sample the entire auditory spatial field beyond the azimuth and elevation axes to determine the extent of the RF of a neuron.

The internal dimensions of the sound booth should be provided.

We have provided the dimensions in the legend of Fig. 1B:

Speakers were positioned 1 m from the center of the chamber, which had internal dimensions of 2 × 2 × 2 m.

Presumably, lines 126 and 128 are the questions referred to in line 137 as "these questions".  It would be easier flow if those lines were put at the end of the paragraph instead of the middle.

Thank you for the suggestion. We have rephrased those sentences as:

…and our recent work showing neural facilitation to repetitive stimuli from nonpreferred locations outside a neuron’s spatial RF (Chen et al., 2025). However, little is known about how auditory cortex neurons represent the large or full spatial field (Middlebrooks and Pettigrew, 1981; Brugge et al., 1994, 1996; Schnupp et al., 2001; Mrsic-Flogel et al., 2005) during active listening.

To address this question, we…

Gao et al. 2015 is not in the reference list

We have included this reference:

24.	L. Gao, K. Kostlan, Y. Wang, X. Wang. Distinct subthreshold mechanisms underlying rate-coding principles in primate auditory cortex. Neuron, 2016, 91(4): 905-919.

Line 613 states that the behavioral paradigm is in Fig 1A but more likely refers to Fig 1D.

We have fixed this error (Fig. 1C is now based on the original Fig. 1D):

Fig. 1C illustrates the behavioral paradigm.

Line 634 refers to Fig 1B and C but more likely mean Fig 1E and F.

We have fixed this error (Figs. 1A, B are now based on the original Figs. 1F, E):

A diagram of one such condition is shown in Figs. 1A, B.

Line 164: should refer that this is a single unit, not 'the receptive field'

We have revised this by adding “of an example unit” as follows:

Figure 1D shows the spatial RF of an example unit, which was…

Figure 2 legend: should stat that it is the receptive field of a single neuron, not of a monkey.

We have added “of an example unit” in the legend of Fig. 1D as follows:

(D) Passive spatial RF of an example unit in one animal (M9X-74).

We have done the same for the legend of Fig. 2C:

(C) Passive spatial RF of an example unit in the other animal (M71V-2522).

Figure 2 legend: C states that the vertical bar represents 200 ms which makes little sense.  Is the y-axis representing 200 spikes? Spkes/sec? spikes/stimulus? Is there supposed to be a horizontal bar that represents 200 ms? Or is it the vertical shaded bar as opposed to a line?

We apologize for this confusion; the reviewer's last interpretation is correct. We have revised the legend of Fig. 1F to read:

The vertical shaded gray bar indicates the 200-ms sound stimulus.

Line 316 should read: "… models were first …"
We have revised it as:

The normalization models were first developed to model visual responses to stimuli of different contrast (Heeger, 1992), and were subsequently…

Reviewer #2: This study recorded single neurons in marmoset auditory cortex while animals performed a spatial Go/No-Go task that required detecting changes in sound location. By comparing behavior and passive listening, the authors show that many neurons exhibit large firing-rate increases at target locations, including locations outside their classical passive receptive fields, effectively creating "temporary" receptive fields during behavior. These effects were strongest in caudal auditory areas, and additional passive intracellular and random spatial-profile recordings revealed widespread suppressive inputs that may underlie this dynamic modulation. A divisive‐normalization model is proposed to explain how behavior selectively enhances responses at behaviorally relevant spatial locations.  It is a potentially interesting study on dynamic, behavior-dependent modulation of spatial tuning across multiple auditory cortical areas. The manuscript is carefully crafted and
analytically rigorous, integrating a demanding behavioral paradigm with full-field spatial receptive-field mapping in marmoset auditory cortex. The analyses are thoughtfully executed, and the dataset is technically solid and comprehensive.
My suggestions to strengthen the work:

We thank the reviewer for their positive assessment of our study, particularly for noting the rigorous analysis and comprehensive dataset. We appreciate your constructive suggestions to further strengthen the manuscript.

1.      The manuscript would benefit from a clearer articulation of its conceptual advance, as the current framing may feel incremental relative to prior work; more explicitly highlighting what fundamentally new insight about dynamic spatial coding emerges from these results would help strengthen the broader impact of the study.

We appreciate the reviewer’s constructive critique regarding the articulation of our conceptual advance. We agree that the previous framing may have obscured the fundamental novelty of the current results relative to prior work. To address this, we have added a paragraph to the end of the Introduction that explicitly highlights the specific contributions of this study:

This is the first study in a non-human primate species that maps the auditory spatial attention effect across the full spatial field. Our findings demonstrated that top-down attention creates temporary spatial receptive fields in otherwise unresponsive neurons, revealing a stronger task-modulation effect in the caudal “where” pathway than in the rostral “what” pathway. Using computational modeling, we proposed that widespread spatial suppression in the auditory cortex is a potential candidate circuit mechanism to account for behavioral modulations of spatial receptive fields, which was validated through novel data analysis, a new stimulus paradigm, and intracellular recordings in awake marmosets.

2.      The interpretation of the observed modulation as "attention" should be clarified. Because a Go/No-Go task inherently engages arousal, reward expectation, motor preparation, and temporal predictability, it is important to more clearly distinguish these factors or explicitly acknowledge their potential contributions to the neural effects.

We appreciate the reviewer raising these important points regarding potential confounding factors such as arousal, reward expectation, motor preparation, and temporal predictability inherent in the Go/No-Go task. We address each of these factors below.

To address the concern regarding temporal predictability, we refer to our control experiments which used stimuli identical to the behavioral task but with the reward omitted (passive listening). These results demonstrate that the enhanced firing rate during the behavioral condition is not due to temporal predictability.

Below is the text and the new Figure 4A (originally Supplementary Figure 2) regarding our control experiment, which we have now moved to the main text to highlight these findings:

To rule out the possibility that firing rate modulation was attributable to stimulus context, we recorded from a subset of neurons during a 'control' condition in which the stimuli were identical to those in the behavioral condition. In the control comparison, the MI for target locations (0.50; Fig. 4A, left) was similar to the passive comparison; of 111 units tested, 37 and 2 had at least one significantly increased and decreased firing rate to targets, respectively. The control MI for backgrounds of 0.26 (Fig. 4A, middle) was larger than for the passive comparison but did not reach significance (p = 0.15); 16 and 5 units had at least one significantly increased and decreased firing rate to backgrounds, respectively. We further separated significantly modulated locations at target locations into three cortical areas (Fig. 4A, right). The differences among three areas were no longer significant compared to the hits/passive condition previously shown (Kruskal-Wallis ANOVA, p = 0.1). This lack of effect is likely partially due to the reduced statistical power of the hits/control comparisons (n = 37 units compared with 86 for the hits/passive comparison), but also due to the increased MI (0.42) in areas R/RT. This suggests that the stimulus order in the control condition may depress firing rates compared to the random stimulus order in the passive condition, particularly in areas R/RT. Together, the enhanced firing rate during the behavioral condition is not due to stimulus order or temporal predictability.

Figure 4. Context-independent but task-performance-dependent firing rate modulations. (A) Firing rates for hits vs. control. Left: Target locations. Gray circles indicate non-significant modulation (n=249 above, 199 below diagonal). Purple and green indicate significantly increased and decreased rates, respectively. Middle: Background locations (n=53, 55). Right: Significant target locations separated by cortical area (shapes).

Furthermore, our additional analysis of false alarm trials—where the marmoset is in a high-arousal state, licks the tube, and expects a reward despite the absence of the target stimulus—addresses the remaining concerns regarding arousal, motor preparation, and reward expectation. Below is the text and Fig. 4D:

While miss trials reflect behavioral performance, relying solely on them has limitations; a miss may simply result from low arousal or inattention. A metric that captures both performance and arousal state is the false alarm rate (i.e., licking in the absence of a target) which indicates high arousal or motor readiness. To distinguish between specific auditory processing and general arousal or motor preparation, we analyzed the relationship between neural modulation (MI) and behavioral sensitivity (d'), which accounts for both hit and false alarm rates. We divided all significantly modulated locations into groups with positive and negative MIs and visualized the relationship between perceptual sensitivity and decision probability (Fig. 4D). If firing rates are positively correlated with task performance (i.e., positive MI aligns with higher d'), the activity likely reflects auditory processing, as the neuron differentiates between a correct detection (hit) and an impulsive error (false alarm) despite similar motor behavior. Conversely, if activity mainly reflects motor preparation or arousal, the neuron would fire whenever the animal licks regardless of accuracy. Our results support the former hypothesis: the median d' of the positive MI group (1.375) was nearly double that of the negative MI group (0.684). Importantly, the median false alarm rate was identical (0.2) for both groups. In summary, enhanced neural responses correlate with task performance rather than non-specific motor preparation or arousal.

Figure 4. Context-independent but task-performance-dependent firing rate modulations. (D) Left: Schematic of signal detection metrics (false alarm, hit rates, criterion) and sensitivity (d'). Right: Scatter plot of d' versus modulation index (MI) for significantly driven targets (n=197). Black crosses indicate medians for positive (n=148) and negative MI groups.

Note that the false alarm rate is the same between the two groups although the hit rate is different. Therefore, the increased firing rate we observed is most likely due to auditory spatial attention rather than factors including arousal, reward expectation, or motor preparation. We have added the details of this analysis to the Methods.

3.      The link between neural activity and behavior could be strengthened by relating modulation strength more directly to performance measures, e.g. by comparing hit versus miss trials or correlating modulation indices with session-level behavioral sensitivity, to demonstrate that the observed neural changes are behaviorally meaningful.

We appreciate this insightful suggestion. We have performed three additional analyses to explicitly strengthen the link between neural activity changes and behavioral performance.

First, we compared the firing rate difference between miss trials (active condition) and the passive condition. Below is the relevant text and Figure 4B from the revised manuscript:

Given that correctly localized sounds increase the firing rate of auditory cortical neurons (i.e., positive MI), we examined neural responses when the animal failed to localize the target (miss trials). Fig. 4B (left) shows that during miss trials, only 63% of target locations exhibited significantly increased firing rates. The median MI was 0.3, which is significantly lower than that of hit trials (0.48; p = 5.9e-04, rank-sum test). For background locations (Fig. 4B, middle), miss trials tended to decrease the firing rate relative to the passive condition (median MI: -0.15). When examining the three cortical areas separately (Fig. 4B, right), the proportion of locations with increased firing rates was substantially lower during misses compared to hits (74%, 68%, and 40% vs. 90%, 88%, and 50%).

Figure 4. Context-independent but task-performance-dependent firing rate modulations. (B) Firing rates for misses vs. passive. Left: Target locations (n=413, 397). Middle: Background locations (n=141, 149). Right: Significant locations separated by cortical area.

Second, as suggested, we directly compared the firing rate differences between hit and miss trials. Below is the relevant text and Figure 4C:

We further directly compared firing rates between hit and miss trials at target locations (Fig. 4C) and found that successful localization elicited higher firing rates (78% above diagonal; median MI = 0.28). Thus, the enhanced firing rate observed during behavior is performance-dependent, occurring primarily during hit rather than miss trials.

Figure 4. Context-independent but task-performance-dependent firing rate modulations. (C) Firing rates for hits vs. misses at target locations (n=435, 300).

Finally, we correlated the modulation indices (MIs) with session-level behavioral sensitivity (d'), as suggested. Below is the relevant text and Figure 4D (referencing the same analysis mentioned in our response regarding false alarm trials):

While miss trials reflect behavioral performance, relying solely on them has limitations; a miss may simply result from low arousal or inattention. A metric that captures both performance and arousal state is the false alarm rate (i.e., licking in the absence of a target) which indicates high arousal or motor readiness. To distinguish between specific auditory processing and general arousal or motor preparation, we analyzed the relationship between neural modulation (MI) and behavioral sensitivity (d'), which accounts for both hit and false alarm rates. We divided all significantly modulated locations into groups with positive and negative MIs and visualized the relationship between perceptual sensitivity and decision probability (Fig. 4D). If firing rates are positively correlated with task performance (i.e., positive MI aligns with higher d'), the activity likely reflects auditory processing, as the neuron differentiates between a correct detection (hit) and an impulsive error (false alarm) despite similar motor behavior. Conversely, if activity mainly reflects motor preparation or arousal, the neuron would fire whenever the animal licks regardless of accuracy. Our results support the former hypothesis: the median d' of the positive MI group (1.375) was nearly double that of the negative MI group (0.684). Importantly, the median false alarm rate was identical (0.2) for both groups. In summary, enhanced neural responses correlate with task performance rather than non-specific motor preparation or arousal.

Figure 4. Context-independent but task-performance-dependent firing rate modulations. (D) Left: Schematic of signal detection metrics (false alarm, hit rates, criterion) and sensitivity (d'). Right: Scatter plot of d' versus modulation index (MI) for significantly driven targets (n=197). Black crosses indicate medians for positive (n=148) and negative MI groups.

4.      Statistical independence concerns should be addressed, as treating each spatial location as an independent data point may inflate significance. Incorporating unit-level summary metrics or mixed-effects approaches would provide more robust population-level conclusions.

We appreciate this important suggestion regarding statistical independence. To address this, we have implemented both mixed-effects models and unit-level summary metrics to ensure robust population-level conclusions.

Below is the relevant text and figure from the revised manuscript demonstrating these analyses:

Averaging the firing rate in hit trials of one session at one target and background location, and treating each value as an independent data point, may violate the assumption of statistical independence. We addressed this concern with two additional analyses. First, to account for repeated measures from the same units, we employed a linear mixed-effects model (Parida et al., 2023) with task condition (hit vs. passive) and spatial location as fixed effects, and unit identity as a random effect. The model formula was: firing rate ~ condition + location + (1 | unit ID). We found a significant main effect of task condition (F = 60.8, p = 9.3e-15), confirming that engagement in the sound localization task robustly modulated firing rates in the auditory cortex independent of spatial tuning preferences (F = 21.2, p = 8.2e-35). Second, we averaged the firing rate in hit trials across all sessions and all target and background locations (Supplementary Fig. 2). These unit-level metrics were nearly identical to the previous location-level metrics (83% and 56%, Fig. 3A, B): 82% and 56% of units exhibited increased firing rates during hit trials at target and background locations, respectively. These results are also consistent with the percentage of units with at least one significantly increased firing rate at target and background locations (79% and 53%). Thus, incorporating the mixed-effects model and unit-level metrics provided robust and consistent population-level conclusions.

Supplementary Figure 2. Attention increases firing rates at the single-unit level.

We have also updated the Methods section to include the details of the mixed-effects model


5.      The boundaries between newly collected behavioral data and previously published passive or intracellular recordings could be more clearly delineated, especially when these datasets are used to support mechanistic interpretations, to avoid confusion about what was measured in behaving animals versus passive conditions.

We appreciate the reviewer pointing this out. To clearly distinguish between the datasets and avoid confusion, we have split the original Figure 5 into two separate figures.

Figure 6 contains data from previously published studies in passively listening animals. We have stated this clearly in both the main Results and the Figure 6 legend:

Here, we reanalyzed spatial RFs published previously (Remington and Wang, 2019) recorded during passive listening.

Figure 6. Firing rate suppression evoked by stimuli from locations outside spatial RFs. All data were previously published in a study of passively listening marmosets (Remington and Wang, 2019).

Figure 7 contains new, unpublished results presented for the first time in this study. We have also stated clearly that these data were collected in passively listening marmosets in both the main Results and the Figure 7 legend:

Using this paradigm, we revealed widespread suppression at off-RF locations that showed near-zero firing rates in passively listening marmosets.

Therefore, we used intracellular recordings to measure membrane potentials in response to different locations in the half spatial field in passively listening marmosets.

Figure 7. Firing rate suppression and membrane potential hyperpolarization evoked by stimuli from locations outside spatial RFs. These data were collected from passively listening marmosets but have not been published previously.

6.      The normalization model, while plausible, remains largely descriptive; constraining it more tightly with empirical data, for example by fitting parameters to reproduce the observed modulation patterns, would make the mechanistic claims more convincing.

We fully agree with the reviewer's suggestion regarding “fitting parameters to reproduce the observed modulation patterns.” We performed this analysis during the initial submission; below is the relevant text from the manuscript:

Figs. 5C–E show the histograms of 66 simulated sessions that matched two experimental constraints. One was that the proportion of attention‑suppressed locations (points below the diagonal in (B), left) lay between 10 % and 30 % (experiment: 21 %). The other one (Supplementary Fig. 3A) was that the ratio of inside-RF locations (scaled passive rate 0.7–1.0 in (B), right) to outside-RF locations (0.2–0.5) exceeded 40 % (experiment: 62 %).

To make this clearer, we have emphasized that our model’s parameters are constrained by experimental data in Figure 5 and in the Figure 5 legend.

Figure 5. (C–E) Distributions of model parameters constrained by the experimental results: (C) Suppression‑field width (σ), (D) Maximum suppression‑field drift, and (E) Maximum allowable attention-field drift.

Reviewer #3: In this study, Chen and colleagues investigated how attention to sound location affects neural activity in the auditory cortex. To this end, marmosets were trained to attend to specific sound locations. The authors found that while most neurons increased their firing rates for target sounds presented inside their RFs, many also showed enhanced responses to sounds outside their RFs, sometimes even exceeding responses at the RF center during passive listening. This off-RF facilitation was especially prominent in the caudal auditory cortex. A computational model explained this effect through widespread neural suppression, which was confirmed by physiological evidence of reduced firing rates and hyperpolarized membrane potentials obtained from intracellular recordings. Overall, the results indicate that behavioral demands expand the neural population involved in processing spatial sound beyond those normally active during passive listening.

The study is well written, methodologically sound, and the number of animals used is appropriate for research involving non-human primates. The results are convincing and clearly discussed. Overall, the study appears ready for publication, with only a few minor comments. This work will be of great interest and is well suited for publication in PLOS Biology.

We thank the reviewer for the positive assessment of our study and for recognizing the significance of our findings regarding the effects of top-down attention on spatial representation in the auditory cortex.

Minor comments:
Figure 1: Please clearly indicate which subfigures illustrate behavioral tasks from previous studies and which are from the current study.

To avoid potential copyright issues regarding the reproduction of schematics from other publications, we have removed the panels illustrating stimulus paradigms from previous studies (original Fig. 1A–C). The revised Figure 1A–C now exclusively illustrates the behavioral task and paradigm used in the current study.

Figure 2: The authors may want to consider using different colors, as the current ones are difficult to distinguish from each other.

We agree that the original figure was difficult to read. In the revised manuscript, we have moved three panels from Figure 2 to Figure 1 (also consistent with suggestions from Reviewer #1) and reorganized the remaining panels. These changes made the distinct panels and data points much clearer and easier to distinguish. Below is the revised Figure 2.

Figure 2. Firing rates increase at locations outside the RFs during spatial discrimination.
